# Dark conditions enhance aluminum tolerance in several rice cultivars via multiple modulations of membrane sterols

**DOI:** 10.1093/jxb/erx414

**Published:** 2017-12-23

**Authors:** Tadao Wagatsuma, Eriko Maejima, Toshihiro Watanabe, Tomonobu Toyomasu, Masaharu Kuroda, Toshiya Muranaka, Kiyoshi Ohyama, Akifumi Ishikawa, Masami Usui, Shahadat Hossain Khan, Hayato Maruyama, Keitaro Tawaraya, Yuriko Kobayashi, Hiroyuki Koyama

**Affiliations:** 1 Faculty of Agriculture, Yamagata University, Tsuruoka, Japan; 2 Graduate School of Agriculture, Hokkaido University, Sapporo, Japan; 3 NARO Agricultural Research Center, Joetsu, Japan; 4 Plant Science Center, RIKEN, Yokohama, Japan; 5 Department of Biotechnology, Graduate School of Engineering, Osaka University, Suita, Osaka, Japan; 6 Tokyo Institute of Technology, Tokyo, Japan; 7 HMD Science and Technology University, Dinajipur, Bangladesh; 8 Faculty of Applied Biological Sciences, Gifu University, Gifu, Japan

**Keywords:** Al tolerance, carotenoid, dark conditions, *HMG* gene, rice, sterol, stigmasterol

## Abstract

Aluminum-sensitive rice (*Oryza sativa* L.) cultivars showed increased Al tolerance under dark conditions, because less Al accumulated in the root tips (1 cm) under dark than under light conditions. Under dark conditions, the root tip concentration of total sterols, which generally reduce plasma membrane permeabilization, was higher in the most Al-sensitive *japonica* cultivar, Koshihikari (Ko), than in the most Al-tolerant cultivar, Rikuu-132 (R_132_), but the phospholipid content did not differ between the two. The Al treatment increased the proportion of stigmasterol (which has no ability to reduce membrane permeabilization) out of total sterols similarly in both cultivars under light conditions, but it decreased more in Ko under dark conditions. The carotenoid content in the root tip of Al-treated Ko was significantly lower under dark than under light conditions, indicating that isopentenyl diphosphate transport from the cytosol to plastids was decreased under dark conditions. *HMG2* and *HMG3* (encoding the key sterol biosynthetic enzyme 3-hydroxy-3-methylglutaryl CoA reductase) transcript levels in the root tips were enhanced under dark conditions. We suggest that the following mechanisms contribute to the increase in Al tolerance under dark conditions: inhibition of stigmasterol formation to retain membrane integrity; greater partitioning of isopentenyl diphosphate for sterol biosynthesis; and enhanced expression of *HMG*s to increase sterol biosynthesis.

## Teaser

Aluminum tolerance of aluminum-sensitive rice was enhanced under darkness by multiple changes in membrane sterols: decreased stigmasterol, increased precursor partitioning for sterols biosynthesis, and increased expression of *HMG* genes.

## Introduction

To adapt to acidic soil environments, plants have developed various strategies to protect root tips from aluminum (Al). The molecular basis and the role of Al sensing and signaling in Al tolerance have been reviewed (Liu *et al.*, 2014).

A few studies have revealed some of the mechanisms by which the composition of plasma membrane (PM) lipids affects Al tolerance. Dysfunction of phosphatidyl phosphohydrolase in an Arabidopsis mutant led to the accumulation of phospholipids in the PM and a decrease in Al tolerance (Kobayashi *et al.*, 2013). The expression level of *PsCYP51* (encoding OBT 14DM in pea, *Pisum sativum* L.) was correlated with Al tolerance, and knocked-down *CYP51A* expression suppressed Al tolerance in Arabidopsis. The decreased expression level of *CYP51* lowered the sterol content and increased the PM permeability in Al-treated root tips (Wagatsuma *et al.*, 2015). Sterols are essential for the maintenance of membrane fluidity and permeability in living cells, and the ion leakage from roots (as an index of membrane integrity) was markedly increased in *cyp51* mutants (Kim *et al.*, 2005). All sterol species in plants affect membrane fluidity, but each one has different effects depending on its structure (Hartmann, 1998). Among all the sterols, sitosterol most strongly restricts the mobility of the surrounding phospholipid acyl chains. The *trans*-oriented double bond at C22 in the side chain of stigmasterol significantly reduces its ordering ability, with an efficiency that can be measured by the reduction in water permeability through the membrane; the order of efficiency is sitosterol>campesterol>>stigmasterol (Schuler *et al.*, 1991). Therefore, higher proportions of stigmasterol lead to greater membrane permeability. To date, there are no reports on the contribution of different sterol species to differences in Al tolerance.

The synthesis of lipids in the PM is under complex regulation, and responds to various environmental conditions other than Al. Seedlings grown under −P pretreatment showed enhanced Al tolerance, which was mainly attributed to a decrease in phospholipids and increase in galactolipids in the PM of root cells (Maejima *et al.*, 2014). Enzymes in sterol biosynthesis pathways show different activities under light and dark conditions. For example, 3-hydroxy-3-methyglutaryl CoA reductase (HMGR, encoded by *HMG*), the key limiting enzyme in phytosterol biosynthesis via the mevalonate (MVA) pathway (Schaller *et al.*, 1995), was induced under dark conditions in Arabidopsis seedlings, except in the roots (Enjuto *et al.*, 1994; Learned, 1996). In rice roots, expression of *HMG2*, which is also involved in sterol biosynthesis, was slightly increased in the dark (Ha *et al.*, 2001). The overexpression of *HMG* in tobacco (*Nicotiana tabacum* L.) increased sterol contents (Schaller *et al.*, 1995). On the basis of those results, we speculated that a dark treatment could confer Al tolerance via increased expression of *HMG*s.

Sterols are isoprenoid-derived molecules that are found in a wide range of organisms including plants (Schaller, 2004). All isoprenoids are biosynthesized from a common precursor, i.e. the five-carbon (C_5_) molecule isopentenyl diphosphate (IPP; see Fig. 7). In plants, IPP is produced via both the MVA and the methylerythritol 4-phosphate (MEP) pathways, although they are localized in different cell compartments, i.e. the cytosol/ER and plastids, respectively. The MEP pathway was discovered only relatively recently (Lichtenthaler *et al.*, 1997); consequently, little is known about the crosstalk between the MEP and MVA pathways, and especially their roles in producing IPP in the roots under dark conditions.

In this study, Al-sensitive rice cultivars showed increased Al tolerance under dark conditions. Therefore, we conducted molecular and physiological analyses to explore the reasons for the difference in Al tolerance between dark-grown and light-grown plants. Changes in the sterol status in the dark led to enhanced Al tolerance of *temperate japonica* rice cultivars. These changes were related to IPP crosstalk between the cytosol and the plastid, and the increased expression of *HMG* genes in the root tip in the dark.

## Materials and methods

### Plant material and growth conditions

We used six cultivars of rice: five *temperate japonica* subpopulations (Rikuu-132, Hidekomochi, Norin-21, Rikuu-20, and Koshihikari) and one *Indica aus* subpopulation (Kasalath) (Xu *et al.*, 2009). Seeds were soaked in tap water with aeration for 24 h at 27 °C in a growth room and germinated under fluorescent white light (80 µmol m^−2^ s^−1^). The germinated seeds were spread on a nylon screen, placed on a container filled with 9 l tap water containing (in mg l^−1^) Ca 8.0, Mg 2.92, K 1.95, and minor quantities of other minerals (P, Fe, Mn, Zn, and Cu) (Khan *et al.*, 2009), and grown for 5 d.

### Analysis for Al tolerance under different illumination conditions

Twelve seedlings of each cultivar were used as a set of seedlings for root growth experiments. Sets of seedlings with similar root length (*ca* 4 cm) were pre-incubated in control solution (pH 4.9; 0.2 mM CaCl_2_) for 6 h. Then, each set was transferred to the Al-toxic solution containing 10 µM AlCl_3_ (+Al) or control solution (−Al). Root elongation was measured after 24 h of incubation and then relative root elongation (+Al/−Al, expressed as a percentage) was calculated as an index of Al tolerance (Khan *et al.*, 2009). Seedlings were kept in the light (L; continuous light; 80 µmol m^−2^ s^−1^), in the dark (D) or in the dark with MVA (1 mM; Sigma-Aldrich, St Louis, MO, USA) and glucose (1 mM) (DMG). The pH of all solutions was maintained at 4.9 by adjusting at 2 h and 18 h after the start of the treatment. The root growth experiment was replicated independently using five sets for L, four sets for D, and two sets for DMG. The same trends were observed in each replicated experiment, and so the results from all experiments were pooled and used to calculate average values and standard errors.

### Visualization of Al accumulation in roots

After treatment with or without Al, whole roots were stained with hematoxylin (0.2% hematoxylin in 0.02% sodium iodide, w/w, pH 4.8) for 15 min as described by Khan *et al.* (2009), and Al accumulation in the root tip was observed under a stereoscope (SMZ-10, Nikon, Tokyo, Japan).

### Real-time qRT-PCR

Total RNA was extracted from 1-cm root tips of cv. Rikuu-132 (R_132_) and cv. Koshihikari (Ko) after 24 h treatment with 0.2 mM CaCl_2_ with or without 10 µM AlCl_3_ (pH 4.9) under L or DMG. Extraction and purification of the total RNA was performed using an RNAqueous column with Plant RNA Isolation Aid (Ambion, Austin, TX, USA). cDNA was synthesized from 1 µg total RNA with a QuantiTech reverse transcription kit (Qiagen, Hilden, Germany). Real-time qRT-PCR using SYBR Green I was carried out with a TP800 thermal cycler (Takara Bio, Shiga, Japan) as described previously (Wagatsuma *et al.*, 2015) using the following gene-specific primers; 5′-GGACGTGGAAAGTCTGTGGT-3′ (sense) and 5′-AACAGCTGAACCAGCAAGGT-3′ (antisense) for *OsHMG2*, and 5′-AAGGCCTTCTTGGATTC-3′ (sense) and 5′-GCAGCAGCTGAATCTCATGT-3′ (anti-sense) for *OsHMG3*. The gene transcript levels were quantified by the standard curve method using a complementary DNA dilution series as described by Bustin *et al.* (2009). The transcript levels of each gene were normalized to that of 18S rRNA.

### Extraction and quantification of sterols and phospholipids in the roots

Sterols were extracted and quantified as described by Suzuki *et al.* (2004). Briefly, the freeze-dried 1-cm root tips of 5-day-old seedlings of three rice cultivars [Kasalath (Ka), Ko, and R_132_] after 24 h treatment with 0.2 mM CaCl_2_ with or without 10 µM AlCl_3_ (pH 4.9) under L or DMG were extracted with CHCl_3_–methanol (1:1), and [25,26,26,26,27,27,27-^2^H_7_]cholesterol was added to the extract as an internal standard. The extract was dried and chromatographed on a silica gel column with hexane–ethyl acetate (2:1) and CHCl_3_–methanol (1:1). The hexane-ethyl acetate eluent was dried, saponified with methanol and 20% KOH, and its eluent with CHCl_3_–methanol was dried. The residue and the debris from extraction were combined and hydrolysed with MeOH and 4 M HCl. These mixtures were extracted with hexane, and the combined hexane layer was dried. The residue was trimethylsilylated and analysed by GC-MS (GC: 6890A, Agilent Technologies, Wilmington, DE, USA; MS: JMS-AM SUN200, JEOL, Tokyo).

Phospholipids were extracted and quantified as described by Khan *et al.* (2009). Briefly, phospholipids were extracted by the modified Bligh and Dyer method [isopropanol: chloroform: H_2_O (1:1:1, v/v/v)]. After purification and dehydration of the extract, phosphorus was quantified by the molybdenum blue spectrophotometric method.

### Extraction and quantification of carotenoids in the root

Carotenoids were extracted and quantified as described by Şükran *et al.* (1998). Briefly, fresh 1-cm root tips of 5-day-old seedlings of two rice cultivars (Ko, R_132_) after 24 h treatment with 0.2 mM CaCl_2_ with or without 10 μM AlCl_3_ (pH 4.9) under L or DMG were extracted with 96% MeOH. The absorbance of the extract was determined at 470, 653, and 666 nm with a spectrophotometer. The carotenoid concentration was calculated as follows: carotenoids (μg ml^−1^)=(1000*A*_470_−2.86chloropyll a−129.2chlorophyll b)/245.

### Statistical analysis

All data were analysed using Fisher’s least significant difference (LSD) test (Fisher, 1958).

## Results

### Aluminum tolerance of rice cultivars and Al accumulation in root tip under different illumination conditions

**Fig. 1.**
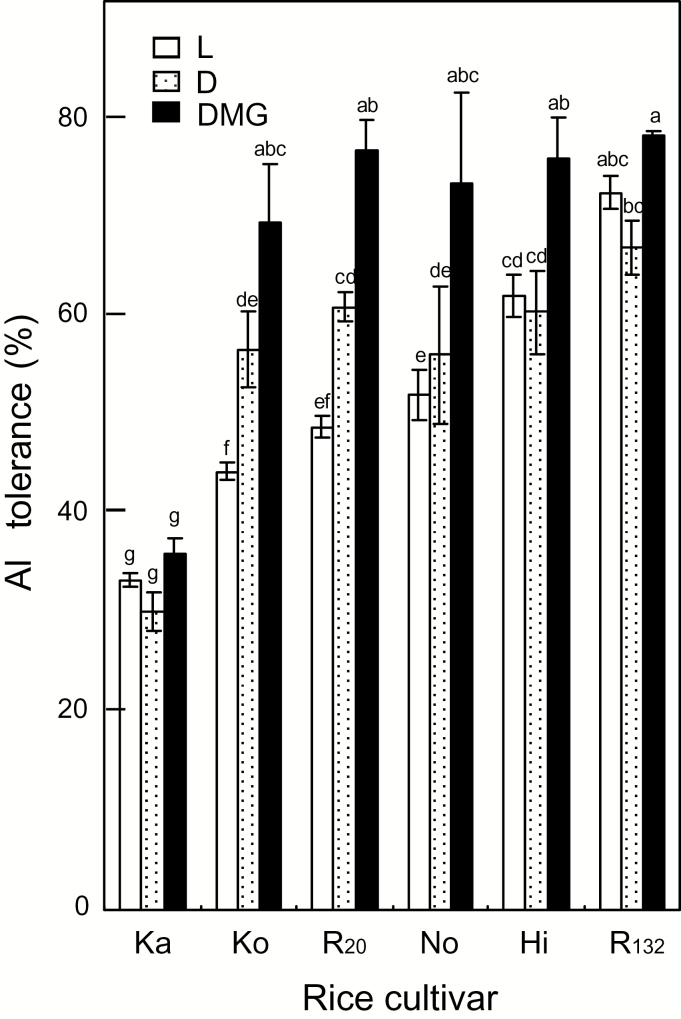
Aluminum tolerance of six rice cultivars under different light conditions. Five-day-old rice seedlings were treated for 24 h with 10 µM AlCl_3_ in the presence of 0.2 mM CaCl_2_ (pH 4.9), and Al tolerance was calculated as the ratio of net elongation of the longest root in the Al treatment to that in the control (0.2 mM CaCl_2_ without Al). Twelve seedlings with similar root length (*ca* 4 cm) were used for one set in Al tolerance analysis. Analysis was carried out independently for five sets for L, four sets for D, and two sets for DMG. L, light; D, dark; DMG, dark in the presence of 1 mM mevalonate and 1 mM glucose. Rice cultivars: Ka, Kasalath; Ko, Koshihikari; Hi, Hidekomochi; No, Norin-21; R_20_, Rikuu-20; R_132_, Rikuu-132. Values are means of independent replicates±standard error. Different letters above bars indicate significant differences (*P*<0.05; Fisher’s LSD).

Under L, the order of Al tolerance among the rice cultivars was as follows: R_132_≥Hidekomochi (Hi)>Norin-21 (No)≥Rikuu-20 (R_20_)≥Ko>Ka (Fig. 1). Under D, the Al tolerance became similar among R_132_, Hi, No, R_20_, and Ko, because of an increase in the Al tolerance of the Al-sensitive cultivars. The Al tolerance of the most Al-sensitive *indica*-type Ka and the most Al-tolerant *japonica*-type R_132_ was unchanged by the different illumination conditions. Addition of MVA and glucose (sterol precursors) to the Al toxic solution further enhanced the Al tolerance of all Al-sensitive *japonica*-type cultivars (Fig. 1). All *japonica*-type cultivars showed similar growth in the Al toxic solution. The most Al-sensitive *japonica*-type cultivar under L, Ko, grew comparably to the most Al-tolerant one, R_132_, under D.

**Fig. 2.**
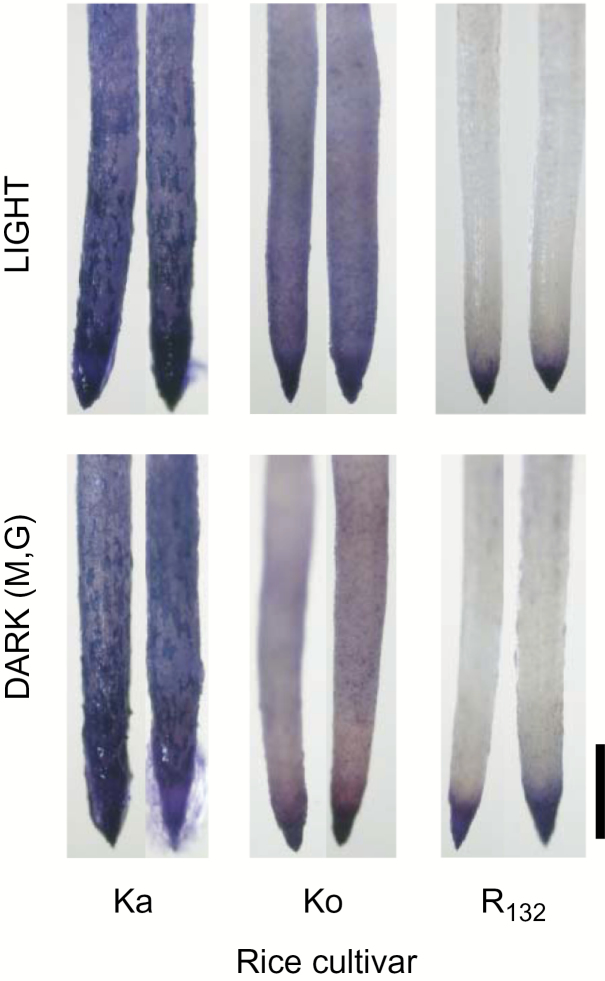
Aluminum accumulation in root tip. Roots of 5-day-old seedlings of three rice cultivars, i.e. Ka (Kasalath), Ko (Koshihikari), and R_132_ (Rikuu-132), were treated for 24 h with 10 μM AlCl_3_ + 0.2 mM CaCl_2_ (pH 4.9) under light (LIGHT) or dark conditions in the presence of 1 mM mevalonate and 1 mM glucose (DARK[M,G]). More than six root tips were used for each cultivar, and two representative tips are shown. Aluminum accumulation was observed by hematoxylin staining; denser purple staining indicates greater Al accumulation. Scale bar: 1 mm.

We compared Al accumulation in the root tip of the three cultivars with significant differences in Al tolerance (Fig. 2). Under L, more Al accumulated in the Al-sensitive cultivars than in R_132_, as judged by the blue color intensity of hematoxylin staining. The blue color in the Ko root tip was lighter under DMG, suggesting that the DMG treatment improved the Al tolerance of Ko by decreasing Al accumulation in the root tip.

### Sterol profile and phospholipid content in the root tip under different illumination conditions

**Fig. 3.**
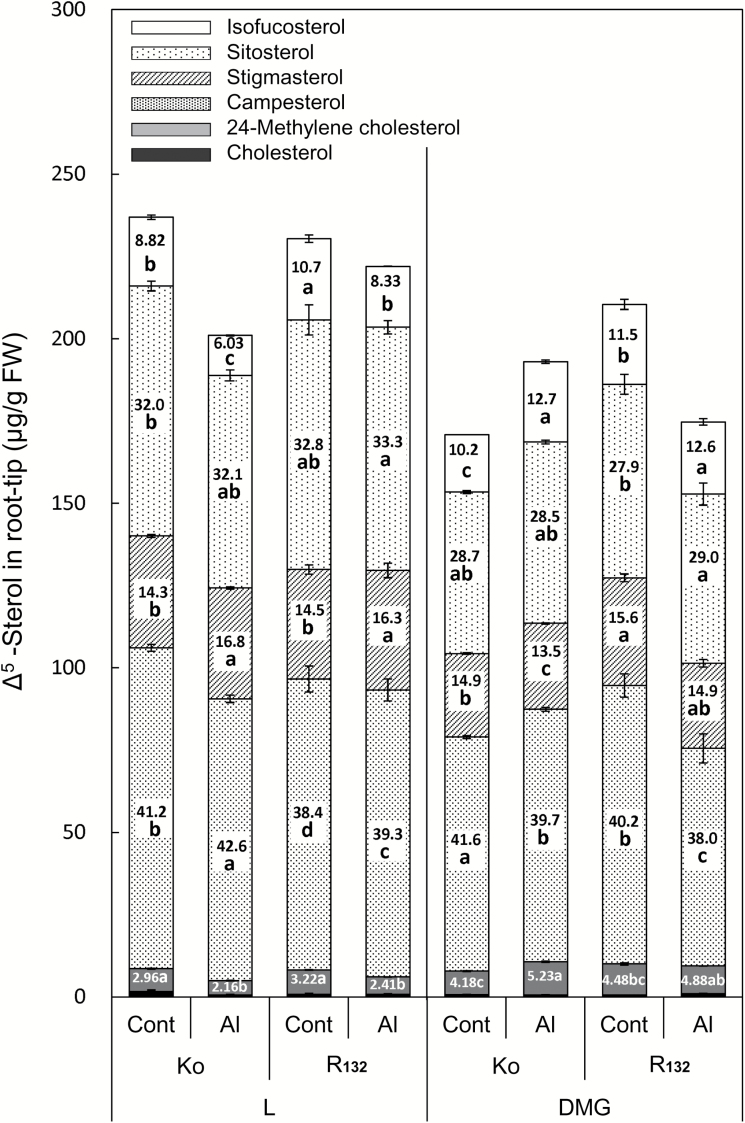
Sterol composition in 1-cm root tip of two rice cultivars (Ko, Koshihikari; R_132_, Rikuu-132) under different illumination conditions. Length of each bar indicates concentration of Δ^5^-sterols (µg g^−1^ FW of root tips); number within each bar indicates relative proportion of each sterol to total Δ^5^-sterols in that treatment (%). As the composition of cholesterol was too low (0.33–0.69%), it is concealed below that of 24-methylene cholesterol. Five-day-old rice seedlings were treated for 24 h with 0.2 mM CaCl_2_ in the presence or absence of 10 µM AlCl_3_ (pH 4.9) under light conditions (L) or dark conditions in the presence of 1 mM mevalonate and 1 mM glucose (DMG). Values are means of three independent replicates±standard error. Different letters in bars indicate significant differences (*P*<0.05; Fisher’s LSD) among the same sterol species in all treatments under the same illumination conditions.

Irrespective of the genotype, Al treatment, or illumination conditions, the major sterol species were sitosterol, stigmasterol, and campesterol (together accounting for 81–92% of total sterols) and the minor sterol species were isofucosterol and 24-methylene cholesterol (together accounting for 8.2–18.2% of total sterols) (Fig. 3). Other sterol species accounted for only small proportions of total sterols: i.e. cholesterol, 0.33–0.69%; cycloartenol, 0.12–0.87%; unidentified sterols, 0.44–0.81% (data not shown).

Under L, the Al treatment caused a greater decrease in the total sterol content in the Al-sensitive Ko than in the Al-tolerant R_132_, while the sterol content was similar in the two cultivars in the absence of Al under L (Fig. 3). The Al treatment also affected the sterol profile. Under L, the proportion of the two major sterol species (stigmasterol and campesterol) out of total sterols was significantly higher in the Al treatment (stigmasterol, 16.3 ± 0.40% in R_132_ and 16.8 ± 0.13% in Ko; campesterol, 39.3 ± 0.10% in R_132_ and 42.6 ± 0.17% in Ko) than in the control (stigmasterol, 14.5 ± 0.12% in R_132_ and 14.3 ± 0.07% in Ko; campesterol, 38.4 ± 0.09% in R_132_ and 41.2 ± 0.28% in Ko) (*P*<0.05; LSD test). The Al-induced increase in the proportion of the major sterol species was slightly greater in the Al-sensitive Ko than in Al-tolerant R_132_ (Fig. 3 and Table 1). Under L, the proportion of sitosterol was unchanged by the Al treatment (32.8 ± 0.44% [control] and 33.3 ± 0.30% [Al treatment] in R_132_ and 32.0 ± 0.12% [control] and 32.1 ± 0.38% [Al treatment] in Ko).

**Table 1.** *Proportional ratios of major sterol species under different conditions (calculated from data shown in* Fig. 3) Al/Cont: proportion of each sterol species to total sterols in Al/that in control. AlDMG/AlL: proportion of each sterol species to total sterols in Al under DMG/that in Al under L.

			Sitosterol	Stigmasterol	Campesterol
Al/Cont	L	R_132_	1.02	1.12	1.02
		Ko	1.00	1.17	1.04
	DMG	R_132_	1.04	0.96	0.95
		Ko	0.99	0.91	0.95
AlDMG/Al_L_		R_132_	0.87	0.91	0.97
		Ko	0.89	0.80	0.94

Under L, the proportion of the two minor sterol species (isofucosterol and 24-methylene cholesterol) out of total sterols was significantly lower in the Al treatment (isofucosterol, 8.33 ± 0.27% in R_132_ and 6.03 ± 0.16% in Ko; 24-methylene cholesterol, 2.41 ± 0.07% in R_132_ and 2.16 ± 0.09b in Ko) than in the control (isofucosterol, 10.7 ± 0.06% in R_132_ and 8.82 ± 0.14% in Ko; 24-methylene cholesterol, 3.22 ± 0.12% in R_132_ and 2.96 ± 0.03% in Ko). The decrease in the proportion of the minor sterol species by the Al treatment was larger in the Al-sensitive Ko than in the Al-tolerant R_132_ (Fig. 3 and Supplementary Table S1 at *JXB* online).

Under DMG, the total sterol content was decreased by the Al treatment in the Al-tolerant R_132_, as under L. However, the total sterol content in the Al-sensitive Ko was increased by the Al treatment (Fig. 3). The effect of the Al treatment on the sterol profile under DMG was the reverse of that observed under L, that is, the proportion of the two major sterol species (stigmasterol or campesterol) out of total sterols was decreased significantly by the Al treatment in both cultivars except for stigmasterol in R_132_ (stigmasterol, 15.6 ± 0.15% in the control and 14.9 ± 0.35% in the Al treatment for R_132_, and 14.9 ± 0.09% in the control and 13.5 ± 0.09% in the Al treatment for Ko; campesterol, 40.2 ± 0.15% in the control and 38.0 ± 0.31% in the Al treatment for R_132_, and 41.6 ± 0.09% in the control and 39.7 ± 0.23% in the Al treatment for Ko). In contrast, the Al treatment increased significantly the proportion of the minor two sterol species except for 24-methylene cholesterol (isofucosterol, 11.5 ± 0.23% in the control and 12.6 ± 0.24% in the Al treatment for R_132_, and 10.2 ± 0.07% in the control and 12.7 ± 0.18% in the Al treatment for Ko; 24-methylene cholesterol, 4.48 ± 0.05b% in the control and 4.88 ± 0.27% in the Al treatment for R_132_, and 4.18 ± 0.08% in the control and 5.23 ± 0.07% in the Al treatment for Ko). The decrease in one of the major sterol species (stigmasterol) (Fig. 3 and Table 1) and the increase in the minor sterol species (Fig. 3 and Supplementary Table S1) by the Al treatment were larger in the Al-sensitive Ko. The proportion of sitosterol out of the total sterol content was slightly increased or similar for the Al treatment in both cultivars (27.9 ± 0.33% in the control and 29.0 ± 0.47% in the Al treatment for R_132_, and 28.7 ± 0.12% in the control and 28.5 ± 0.06% in the Al treatment for Ko).

**Fig. 4.**
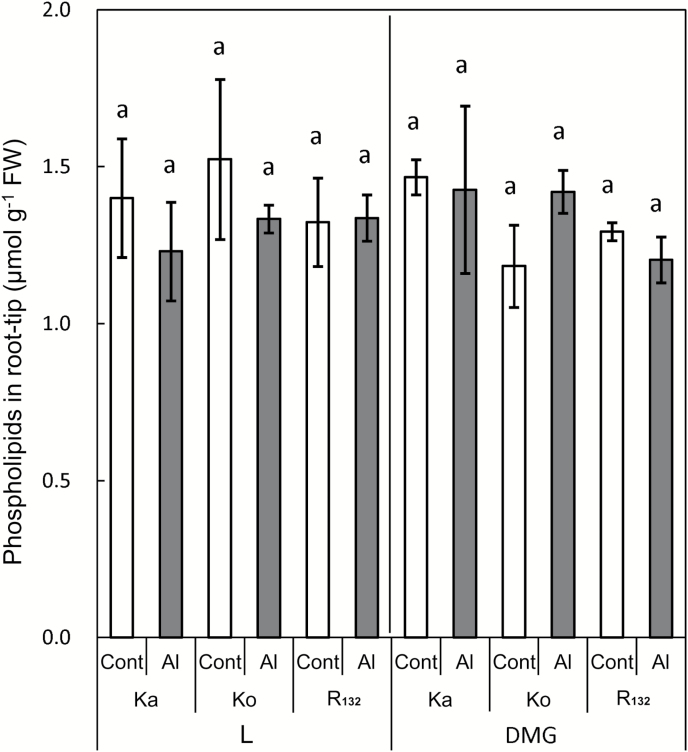
Phospholipids content in 1-cm root tip of three rice cultivars with or without Al treatment under different illumination conditions (μmol g^−1^ FW). Five-day-old rice seedlings were treated for 24 h with 0.2 mM CaCl_2_ in the presence or absence of 10 μM AlCl_3_ (pH 4.9) under light (L) or dark conditions in the presence of 1 mM mevalonate and 1 mM glucose (DMG). Ka, the most Al-sensitive *indica aus* cv. Kasalath; Ko, Al-sensitive *japonica* cv. Koshihikari; R_132_, Al-tolerant *japonica* cv. Rikuu-132. Values are means of independent replicates±standard error. The same letters above bars indicate non-significant differences (*P*<0.05; Fisher’s LSD).

The phospholipid content in the root tip did not differ significantly among the three rice cultivars, i.e. Ka, Ko, and R_132_, irrespective of the Al treatment and illumination conditions (Fig. 4).

### Carotenoid content in root tip under different illumination conditions

**Fig. 5.**
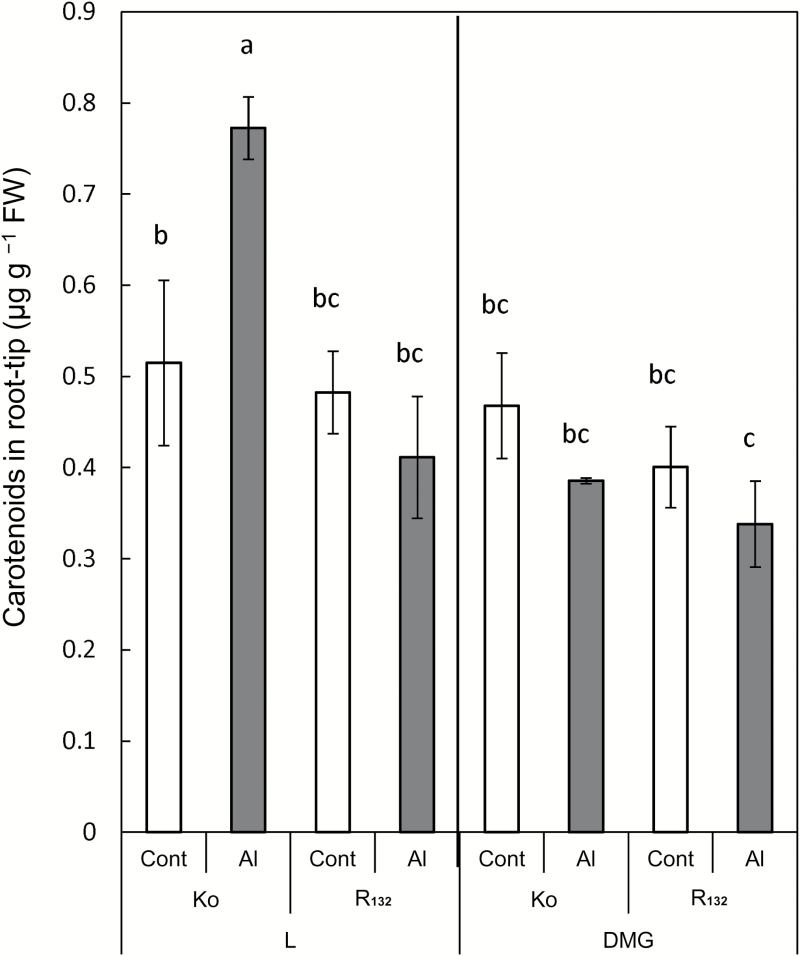
Carotenoid content in 1-cm root tip of two rice cultivars under different illumination conditions. Ko, Koshihikari; R_132_, Rikuu-132; L, light conditions; DMG, dark conditions in the presence of 1 mM mevalonate and 1 mM glucose. Values are means of independent replicates±standard error. Letters (a–c) above error bars indicate significant differences (*P*<0.05; Fisher’s LSD).

To explore the crosstalk between the MVA and MEP pathways under different illumination conditions or Al treatment, we measured the carotenoid content as a measure of IPP transport from the cytosol to the plastids, although plastids contain not only carotenoids but also plastoquinone, monoterpenes, diterpenes, GAs, phytols, and other products (see Fig. 7). Under L, the Al treatment slightly decreased the carotenoid content in the root tip of the Al-tolerant R_132_, but markedly increased that in the root tip of Al-sensitive Ko (Fig. 5). Under D, the carotenoid content in the root tip was slightly decreased by the Al treatment in both R_132_ and Ko.

### Transcript levels of *HMG2* and *HMG3* in root tip under different illumination conditions

**Fig. 6.**
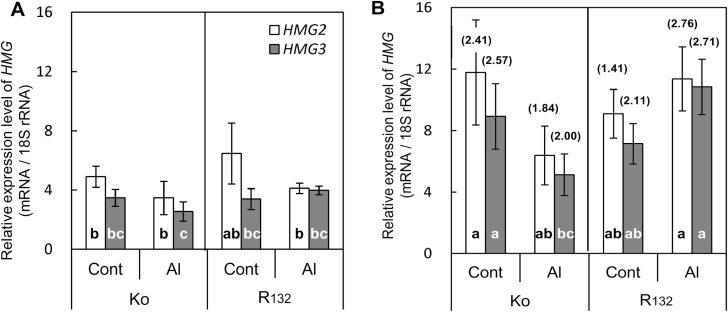
Relative transcript levels of *HMG2* and *HMG3* in 1-cm root tip of two rice cultivars under different light conditions. Five-day-old rice seedlings were treated for 24 h with 0.2 mM CaCl_2_ under L (light conditions) (A) or DMG (dark conditions with 1 mM mevalonate and 1 mM glucose) (B). Total RNA was extracted from frozen 1-cm root tips and used for real-time qRT-PCR. Relative transcript levels of *HMG*s were normalized to that of 18S rRNA (internal control). Numbers in parentheses indicate relative transcript level of each *HMG* under DMG to that under L. Rice cultivars: Ko, Koshihikari; R_132_, Rikuu-132. Values are means of three independent replicates±standard error. Different letters in bars indicate significant differences (*P*<0.05; Fisher’s LSD) among all treatments.

Among the *HMG* genes, both *HMG2* and *HMG3* are thought to be related to sterol biosynthesis although their functions are different (Ha *et al.*, 2001; Ohyama *et al.*, 2007). We measured the mRNA levels of these genes. Under L, both *HMG*s generally showed slightly higher transcript levels in the Al-tolerant R_132_ root tips than in the Al-sensitive Ko root tips, irrespective of the Al treatment. Their transcript levels were generally decreased by the Al treatment (Fig. 6A). The transcript levels of both *HMG*s were higher under DMG than under L (1.41–2.76 times higher) (Fig. 6B). The Al treatment decreased *HMG* transcript levels in the Al-sensitive Ko, but increased them in the Al-tolerant R_132_. Irrespective of the illumination conditions, the transcript level of *HMG2* was always higher than that of *HMG3*.

## Discussion

### New finding that enhanced Al tolerance of rice cultivars in the dark is not related to changes in phospholipids in the root tip

Several studies have focused on the effects of different ions in the rhizosphere (OH^－^, H^＋^, Ca^2＋^, Mg^2＋^, phosphate, SO_4_^2－^) on Al tolerance (Tanaka *et al.*, 1987). However, none has focused on the effects of aboveground conditions on Al tolerance. Here, we have shown for the first time that the Al tolerance of Al-sensitive *temperate japonica* cultivars was enhanced under darkness, especially in the presence of the precursors MVA and glucose (under DMG; Fig. 1). Aside from genetic engineering techniques, no other methods have successfully produced rice lines with enhanced Al tolerance. Dark or DMG conditions did not retard net root elongation in the absence of Al, and the net root elongation in the Al treatment under D or DMG was considerably increased in the most Al-sensitive *temperate japonica* cultivar Ko, compared with that under L (*ca* 60% increase under DMG) (Supplementary Table S2). These results indicate that the increase in Al tolerance under DMG is not an artefact of the calculation of the relative value for Al tolerance, but reflects an actual increase in root elongation under DMG.

The phospholipid content in the root tip did not differ significantly among the three rice cultivars, irrespective of the Al treatment and the illumination conditions (Fig. 4). The weaker negative charge associated with lower phospholipid content in the PM has been reported as an effective Al-tolerance mechanism in rice, Arabidopsis, and tobacco (Khan *et al.*, 2009; Kobayashi *et al.*, 2013, Maejima *et al.*, 2014; Wagatsuma *et al.*, 2015; Zhang *et al.*, 2016). However, we found that the phospholipid content did not contribute to the differences in Al tolerance among cultivars in this study. Greater permeation of Al through the PM into the cytoplasm is suggested to result not only from increased phospholipid content in the PM, but also from increased contents of other lipid species without a negative charge, such as sterols, which affects the permeability of the PM.

### Enhanced Al tolerance of Al-sensitive Ko in the dark is due to quantitative and qualitative changes in sterols and regulated partitioning of IPP to plastids

The lower Al accumulation in the root tip of the Al-tolerant cultivar under L (Fig. 2) is consistent with the results of a previous study (Khan *et al.*, 2009). Under DMG, the Al accumulation in the root tip was remarkably decreased only in the most Al-sensitive *temperate japonica* cultivar Ko. There are no previous reports of a decrease in Al accumulation in the root tip of rice under DMG.

Several studies have isolated and characterized Al tolerance genes from rice (Liu *et al.*, 2014). However, fewer studies have focused on Al tolerance mechanisms or genes related to lipid metabolism.

Under L, the total sterol content in the Al treatment was higher in the Al-tolerant R_132_ than in the Al-sensitive Ko (Fig. 3). Under DMG, the increase in Al tolerance in Al-sensitive Ko was accompanied by an increase in the total sterol content and the relatively greater decrease in Al accumulation in the root tip, compared with that in the root tip of the Al-tolerant R_132_ in the Al treatment (Figs 1–3). Therefore, our results indicate that higher Al tolerance is related to higher sterol content and lower Al accumulation in the root tip. These points agreed with the results of other studies on pea (*P. sativum*), triticale (×*Triticosecale* Wittmark cv. Currency), maize (*Zea mays*), wheat (*Triticum aestivum*), sorghum (*Sorghum bicolor*), rice, and Arabidopsis (Khan *et al.*, 2009; Wagatsuma *et al.*, 2015).

**Fig. 7.**
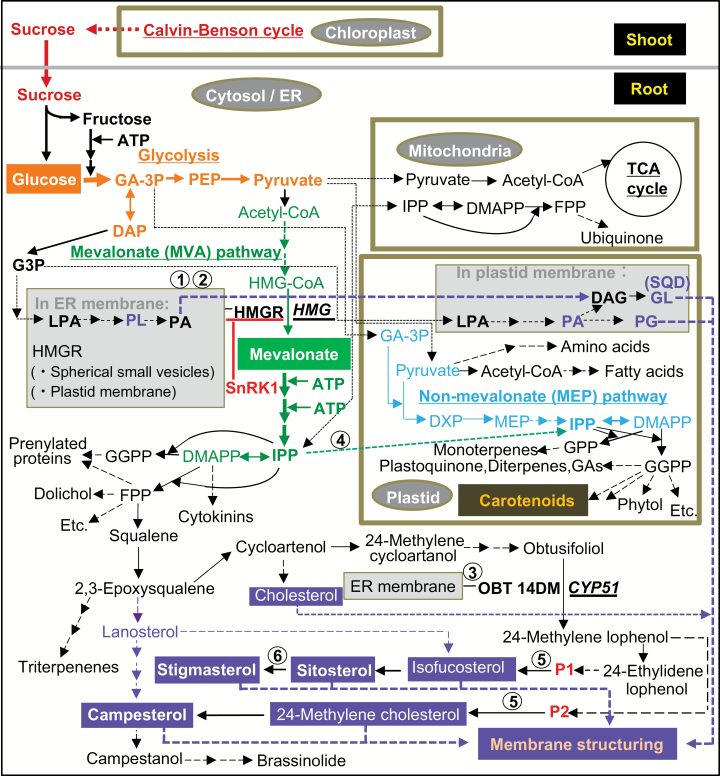
Model for biosynthesis of sterols and other isoprenoids in rice roots. Two independent pathways produce IPP, the precursor of all isoprenoids: the cytosolic acetate/mevalonate (MVA) pathway and the plastidial non-MVA methylerythritol 4-phosphate (MEP) pathway. Figure was compiled by reference to Fig. 1 of Fujioka *et al.* (1997), Fig. 1 of Rodríguez-Concepción *et al.* (2004), and Fig. 1 of Wang *et al.* (2012). Thicker arrows or bold letters in cytosol indicate presumed high concentrations of metabolites under dark conditions with mevalonate and glucose supplied (this study; see Discussion). Glycolysis, MVA, and MEP pathways are shown in orange, green, and blue letters, respectively. Purple dashed arrow shows biosynthetic pathway to phytosterol via lanosterol in dicots. ①, localization of HMGR at endoplasmic reticulum (ER) membrane and partially at spherical small vesicles (Leivar *et al.* 2005); ②, localization of HMGR at plastid membrane (Wong *et al.* 1982); ③, localization site of OBT 14DM at ER membrane (Bak *et al.* 1997); ④, greater inhibition of cytosolic IPP partitioning from the cytosol to the plastid by Al treatment in Al-sensitive Ko under DMG than under L; ⑤, Δ^5,7^-sterol Δ^7^-reductase encoded by *DWARF5*; ⑥, sterol-Δ^22^-desaturase encoded by *CYP710A1*; DAP, dihydroxyacetone phosphate; DMAPP, dimethylallyl diphosphate; DXP, 1-deoxy-D-xylulose-5-phosphate; FPP, farnesyl diphosphate; GA-3P, glyceraldehyde-3-phosphate; GAs, gibberellins; GGPP, geranylgeranyl diphosphate; G3P, glycerol 3-phosphate; GL, galactolipids; GPP, geranyl diphosphate; *HMG*, 3-hydroxy-3-methylglutaryl-CoA reductase encoding gene; HMGR, HMG reductase; IPP, isopentenyl diphosphate; LPA, lysophosphatidate; MEP, methylerythritol-4-phosphate; P1, stigmasta-5,7,Z-24(24^1^)-trien-3β-ol; P2, ergosta-5,7,24(24^1^)-trien-3β-ol; PEP, phosphoenol pyruvate; PG, phosphatidylglycerol; PL, phospholipids; SnRK1, sucrose non-fermenting 1-related kinase 1; SQD, sulfoquinovosyldiacylglycerol.

Campestanol, P_1_ (stigmasta-5,7,Z-24(24^1^)-trien-3β-ol) and P_2_ (ergosta-5,7,24(24^1^) -trien-3β-ol) (Fig. 7) were far lower than those of the three major sterols and the two minor sterol species detected in this study (Fujioka *et al.*, 1997; Kim *et al.*, 2005). Therefore, the changes in the sterol profile can be discussed based on the fate of these five sterol species. In normal light conditions, the proportional ratio (Al/Cont) of the two major sterols (sitosterol and campesterol) in the Al-tolerant R_132_ was almost 1.0. The proportional ratio of stigmasterol was increased to 1.12 in the Al-tolerant R_132_ and 1.17 in the Al-sensitive Ko (Table 1). Under DMG, however, the proportional ratio of stigmasterol was decreased in both cultivars (0.96 in Al-tolerant R_132_ and 0.91 in Al-sensitive Ko), and the decrease was greater in the Al-sensitive Ko. There was a greater decrease in the ratio of the proportion of stigmasterol to total sterols in the Al treatment under DMG to that under L (Al_DMG_/Al_L_) in the Al-sensitive Ko than in the Al-tolerant R_132_ (0.91 in Al-tolerant R_132_ and 0.80 in Al-sensitive Ko). Stigmasterol has no ability to reduce membrane permeabilization because of the *trans*-oriented double bond at C22 in its side chain (Schuler *et al.*, 1991; Hartmann, 1998).

The initial Δ^5^-sterols, i.e. isofucosterol and 24-methylene cholesterol, are formed from P_1_ and P_2_, respectively (Fig. 7), in a reaction catalysed by Δ^5,7^-sterol Δ^7^-reductase encoded by *DWARF5* (Choe *et al.*, 2000; Benveniste, 2002) (⑤ in Fig. 7). Formation of stigmasterol from sitosterol is catalysed by sterol-Δ^22^-desaturase encoded by *CYP710A1* (Morikawa *et al.*, 2006). We suggest that the reduced proportion of stigmasterol in the Al-sensitive Ko under DMG may be due to inhibition of sterol-Δ^22^-desaturase (⑥ in Fig. 7) by Al that accumulated to moderately high level in the Al-sensitive Ko (Fig. 2).

The proposed effects of Al on sterol synthesis, based on the data obtained in this study, can be summarized as follows. Under L, Al inhibited the activity of Δ^5,7^-sterol Δ^7^-reductase, especially in the Al-sensitive Ko, as shown in the sterol profiles in Figs 3 and 7 and Table 1 (decreased isofucosterol and increased stigmasterol in the Al treatment). Under D, Al treatment inhibited the activity of sterol-Δ^22^-desaturase, especially in the Al-sensitive Ko as shown in Figs 3 and 7 and Table 1 (increased isofucosterol and decreased stigmasterol in the Al treatment). The higher Al accumulation in the Al-sensitive Ko (Fig. 2) supports these results. The Al-sensitive Ko may have greater potential to change the Al-targeted enzyme from Δ^5,7^-sterol Δ^7^-reductase to sterol-Δ^22^-desaturase to enhance Al tolerance under D. This is promising information for a new strategy to generate Al-tolerant rice lines, although its detailed mechanisms are unknown. The most Al-sensitive *indica aus*, Ka, showed the largest increase in the proportion of stigmasterol out of total sterols in response to Al, irrespective of the illumination conditions: 20.5 ± 0.26% in the Al treatment and 16.2 ± 0.27% in the control (*P*<0.01; *t*-test) under L, and 20.5 ± 0.73 in the Al treatment and 14.0 ± 0.18% in the control under DMG (*P*<0.01; *t*-test) (Supplementary Fig. S1). This result suggests that the same mechanisms operate in the most Al-sensitive *indica aus*, Ka, as in the two *temperate japonica* cultivars.

Under dark conditions, instant activation of the MVA pathway provides increased amounts of precursors for the biosynthesis of sterols, which are required for growth in search of light (Rodríguez-Concepción, 2006). The pivotal MVA intermediate IPP is transported to the plastid for the production of carotenoids under dark conditions (Park *et al.*, 2002). An active MEP pathway has been found in some non-photosynthetic tissues such as roots (Walter *et al.*, 2002; Hampel *et al.*, 2005; Seemann *et al.*, 2006; Goldwasser *et al.*, 2008; López-Ráez *et al.*, 2008; Kohlen *et al.*, 2011; Abe *et al.*, 2014). Although active crosstalk of cytosolic IPP from the MVA pathway has been reported between the cytosol and the plastid, there is no information on IPP crosstalk in the roots under different illumination conditions. In the Al-sensitive Ko, carotenoid production was inhibited by Al treatment under DMG (Fig. 5), although greater carotenoid accumulation occurred in the Al treatment under L. Carotenoids are not the only plastidial compounds biosynthesized in the MEP pathway, but their fate is a measure of the crosstalk of cytosolic IPP between the cytosol and the plastids (④ in Fig. 6). These results suggested that the Al-sensitive Ko was able to shut down the cytosolic IPP transporter system under Al treatment in DMG to supply more IPP for increased sterol biosynthesis.

### Enhanced expression of *HMG*s and its relationship with sterol biosynthesis in the rice root tip under DMG

We detected *HMG1* (data not shown), but this gene is probably a pseudogene and is not a functional gene in *temperate japonica* rice (Nelson *et al.*, 1994).

The transcript levels of *HMG* genes, especially *HMG2*, were slightly higher in the whole roots of dark-grown seedlings than in those of light-grown seedlings of *temperate japonica* rice (Ha *et al.*, 2001). The *HMG* transcription patterns and levels detected in rice root tips in this study were consistent with the results of Ha *et al.* (2001). The transcript levels of *HMG* genes under DMG were 1.41–2.76 times higher than those under L irrespective of the rice genotype or the presence of Al (Fig. 6). The transcript level of *HMG2* was always higher than that of *HMG3*. Rice *HMG2* is homologous to Arabidopsis *HMG1*, which is a sterol biosynthesis housekeeping gene (Ha *et al.*, 2001). There is no direct evidence for the function of *HMG3* in rice, but its encoded product is thought to be involved in both sterol biosynthesis and triterpenoids biosynthesis. Previous reports have shown that MVA in the medium is incorporated rapidly into sterols (Adler and Kasprzyk, 1975; Atallar *et al.*, 1975). In the present experiment, therefore, we added 1 mM MVA to the medium under D to supply supplementary substrate for sterol biosynthesis (Fig. 7).

Under L, the total sterol content in the root tip was generally parallel to the transcript levels of *HMG*s (Figs 3 and 6A). In the Al treatment, the transcript levels of *HMG*s and the total sterol content were lower in the Al-sensitive Ko than in the Al-tolerant R_132_. The lower total sterol content in the Al-sensitive Ko than in the Al-tolerant R_132_ in the Al treatment (Fig. 3) is proposed to result from the greater inhibition of HMGR activity resulting from the higher concentration of root tip Al (Fig. 2) and the slightly lower transcript levels of *HMG*s (Fig. 6). Under DMG, however, the sterol content in the root tip was not parallel to the transcript levels of *HMG*s (Figs 3 and 6B). One of the reasons for this discrepancy may be the post-translational control of HMGR by sucrose non-fermenting (SNF)-1 related protein kinase-1 (SnRK1), which inactivates HMGR by phosphorylation of Ser-577 (Halford *et al.*, 2003). Further studies are required to clarify the roles of such post-translational regulation. Another possible reason for this discrepancy may be the unknown lipid homeostatic mechanisms observed in several previous studies (Wang *et al.*, 2014; Wagatsuma *et al.*, 2015; Zhang *et al.*, 2016). That is, under normal growth conditions, there was no increase in monogalactosyldiacylglycerol synthase (MGD) in the leaves of tobacco overexpressing *MGD* (Wang *et al.*, 2014; Zhang *et al.*, 2016), nor was there a decrease in sterol content in Arabidopsis plants with knocked-down expression of *CYP51* (which encodes obtusifoliol 14α-demethylase, the enzyme converting obtusifoliol to 24-methylene lophenol, the precursor of Δ^5^-sterols) (Kushiro *et al.*, 2001; Wagatsuma *et al.*, 2015).

Although the expression levels of *HMG*s under DMG were almost double those under L (Fig. 6B), the total sterol content in all root tips was lower under DMG than under L (72.2–96.1% of that under L) (Fig. 3). The light conditions affect many aspects of metabolism, especially sugar-related metabolism. Therefore, the differences in sterol contents under the different illumination conditions reflected natural changes in metabolism. The greater decrease in the total sterol content in Ko under DMG (72.2% of that under L, Fig. 3) than in R_132_ under DMG (90.9% of that under L, Fig. 3) in the control may be due to a larger decrease in sucrose translocation from the shoot to the root tip under dark conditions.

In this study, we found that the Al-sensitive *temperate japonica* rice cultivars showed enhanced Al tolerance under dark conditions. Changes in phospholipids were not the determinant of increased Al permeation into the cytoplasm or differences in Al tolerance among rice cultivars under Al treatment. The results of the study suggest that the Al-sensitive *temperate japonica* rice cultivar had the following control mechanisms in the Al treatment under dark conditions: (i) inhibition of sterol-Δ^22^-desaturase, which decreased the production of stigmasterol; (ii) inhibition of cytosolic IPP transport to the plastid, which increased the supply of the precursor for sterol biosynthesis; and (iii) enhanced expression of *HMG*s, which increased sterols biosynthesis. These findings have identified new targets for the generation of new Al-tolerant plants.

## Supplementary data

Supplementary data are available at *JXB* online.

Table S1. Proportion of minor sterol species under different conditions.

Table S2. Net root elongation of 5-day-old rice cultivars under different illumination conditions.

Fig. S1. Sterol contents in 1-cm root tip of rice cv. Ka under different illumination conditions.

Supplementary Tables S1-S2 and Figure S1

## Abbreviations:

CYP51: cytochrome P450 encoding gene
HMG: 3-hydroxy-3- methylglutaryl CoA reductase (HMGR) encoding gene
IPP: isopentenyl diphosphate
MEP: methylerythritol-4-phosphate
MVA: mevalonate
OBT 14DM: obtusifoliol 14α-demethylase.
