# Supplementary material for: Dark conditions enhance aluminum tolerance in several rice cultivars via multiple modulations of membrane sterols
Source: J Exp Bot. 2017 Dec 23;69(3):567–77. doi: 10.1093/jxb/erx414 (PMC5853495; doi:10.1093/jxb/erx414)
Supplement: Supplementary Tables S1-S2 and Figure S1 [file erx414_suppl_supplementary_tables_s1-s2_and_figure_s1.pdf]

Supplementary Table S1

Proportional ratios of minor sterol species under different conditions (calculated from data shown in Figure 3)

			Isofucosterol	24-Methylene cholesterol
Al/Cont	L	R_{132}	0.78	0.75
		Ko	0.68	0.73
	DMG	R_{132}	1.10	1.09
		Ko	1.25	1.25
Al_{DMG}/Al_{L}		R_{132}	1.51	2.02
		Ko	2.11	2.42

- Al/Cont: proportion of each sterol species to total sterols in Al/that in control
- Al_{DMG}/Al_{L}: proportion of each sterol species to total sterols in Al under DMG/that in Al under L

Supplementary Table S2

Net root elongation of 5-day-old seedlings of rice cultivars in different illumination and medium conditions during 24 h of treatment (mean of 7-10 replicates ± standard error). Number in parentheses is relative root elongation (net elongation in treatment relative to that under light conditions in the same treatment). M, G: 1 m*M* mevalonate and 1 m*M* glucose in the medium.

Cultivar	Treatment	Net root elongation (cm/24h)		
		Light (L) (L=100)	Dark (D) (D/L)	Dark, M, G (DMG) (DMG/L)
R_{132}	Control	1.84 ±0.037 (100)	2.24 ±0.048 (121.7)	1.96 ±0.070 (106.5)
	Al	1.31 ±0.032 (100)	1.47 ±0.086 (112.2)	1.51 ±0.045 (115.3)
Ko	Control	1.78 ±0.051 (100)	2.26 ±0.133 (127.0)	1.93 ±0.175 (108.4)
	Al	0.80 ±0.052 (100)	1.27 ±0.150 (158.8)	1.32 ±0.230 (165.0)
Ka	Control	2.93 ±0.060 (100)	3.14 ±0.087 (107.2)	2.98 ±0.045 (101.7)
	Al	0.92 ±0.034 (100)	0.94 ±0.046 (102.2)	1.05 ±0.030 (114.1)

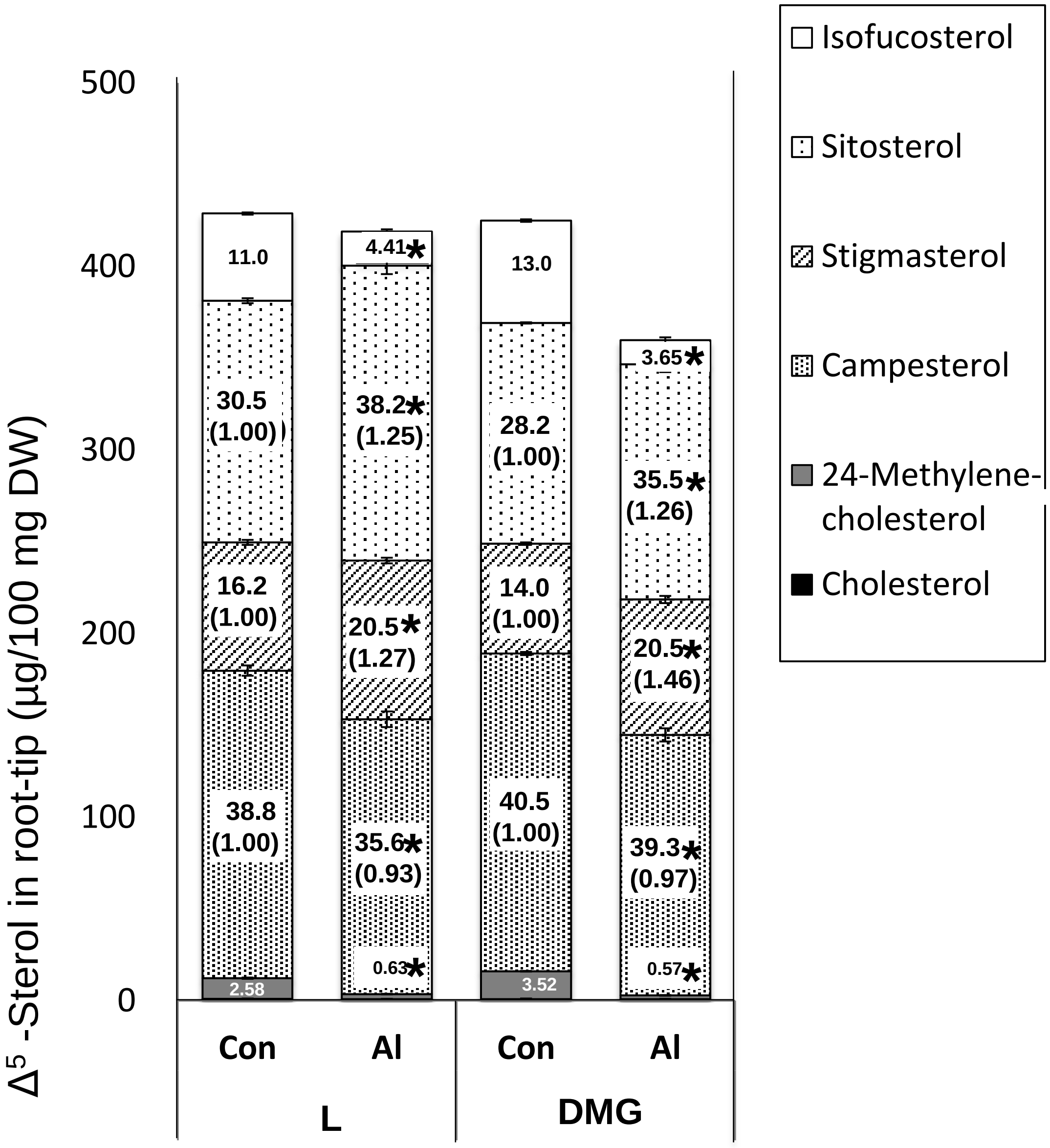

Supplementary Fig. S1

Sterol composition in 1-cm root-tip portion of rice cv. Ka under different illumination conditions. Length of each sterol bar shows concentration of sterols (μg/100 mg DW); number within each sterol bar shows proportion of each sterol species out of total sterols in the same treatment; number in parentheses shows ratio of proportion of each sterol species in the control to that in the Al treatment (%). As the composition of cholesterol was too low, it hides below that of 24-methylene cholesterol. Five-day-old rice seedlings were treated for 24 h with 0.2 m*M* $CaCl_2$ in the presence or absence of 10 μ*M* $AlCl_3$ (pH 4.9) under light conditions (L) or in the dark conditions with 1 m*M* mevalonate and 1m*M* glucose (DMG). Values are means of three independent replicates ± standard error. Asterisk in the right side of the proportion of each sterol species indicates significant difference ($P < 0.01$; t-test) between control and Al treatment under the same illumination conditions.
